# Potassium silica nanostructure improved growth and nutrient uptake of sorghum plants subjected to drought stress

**DOI:** 10.3389/fpls.2024.1425834

**Published:** 2024-07-17

**Authors:** Khadiga Alharbi, Ghalia S. H. Alnusairi, Taghreed S. Alnusaire, Suliman M. S. Alghanem, Ibtisam Mohammed Alsudays, Abdullah Alaklabi, Mona H. Soliman

**Affiliations:** ^1^ Department of Biology, College of Science, Princess Nourah bint Abdulrahman University, Riyadh, Saudi Arabia; ^2^ Department of Biology, College of Science, Jouf University, Sakaka, Saudi Arabia; ^3^ Department of Biology College of Science, Qassim University, Burydah, Saudi Arabia; ^4^ Department of Biology, Faculty of Science, University of Bisha, Bisha, Saudi Arabia; ^5^ Botany and Microbiology Department, Faculty of Science, Cairo University, Giza, Egypt; ^6^ Biology Department, Faculty of Science, Taibah University, Al-Sharm, Yanbu El-Bahr, Yanbu, Saudi Arabia

**Keywords:** crop resilience, potassium nanosilicate, drought stress, nutrient uptake, silica - sulfuric acid

## Abstract

**Introduction:**

Recent advancements in nanotechnology present promising opportunities for enhancing crop resilience in adverse environmental conditions.

**Methods:**

In this study, we conducted a factorial experiment to investigate the influence of potassium nanosilicate (PNS) on sorghum plants exposed to varying degrees of drought stress A randomized complete block design with three replications was employed to subject the sorghum plants to different drought conditions. The three levels of stress were designated as non-stress (NS at -0.03 MPa), moderate stress (MD at -0.6 MPa), and severe stress (SD at -1.2 MPa). The plants were administered PNS at concentrations of 0 mM (control), 3.6 mM Si, and 7.2 mM Si.

**Results and discussion:**

As drought stress intensified, we observed significant reductions in multiple plant parameters, including height, fresh weight, dry weight, leaf number, stem diameter, cluster length, seed weight, and nutrient uptake, with the most pronounced effects observed under SD conditions. Interestingly, nitrogen (N) and potassium (K) levels exhibited an increase under drought stress and PNS application, peaking at MD, alongside Si concentrations. Notably, PNS application facilitated enhanced nutrient uptake, particularly evident in the significant increase in nitrogen concentration observed at 3.6 mM PNS. Furthermore, the application of PNS significantly enhanced the fresh weight and nutrient concentrations (notably K and Si) in sorghum seeds under drought stress, despite varying statistical significance for other nutrients. These findings shed light on the mechanisms through which PNS exerts beneficial effects on plant performance under drought stress. By elucidating the complex interactions between PNS application, drought stress, and plant physiology, this study contributes significantly to the development of sustainable agricultural practices aimed at bolstering crop resilience and productivity in water-limited environments.

## 1 Introduction

Drought stress is a major abiotic challenge that significantly affects agricultural productivity, affecting large areas of the world’s agricultural land and limiting crop growth and performance (Wang et al., 2023). One strategy to alleviate the impact of drought is through the modification of plant nutrients to better withstand drought stress. Imbalances in nutrients can exacerbate soil degradation, leading to reduced moisture retention and nutrient availability, thereby affecting plant health (Silver et al., 2021). Given the detrimental effects of traditional chemical fertilisers on the environment and food quality, researchers have increasingly focused on novel approaches. Among these, nanofertilizers have gained attention for their ability to enhance soil fertility, support plant nutrition, and crucially improve water use efficiency (Jakhar et al., 2022).

Nanoparticles represent a promising avenue in agriculture, albeit still in its nascent stages of global adoption. Positioned at the intersection of interdisciplinary and cutting-edge technology, nanotechnology has emerged as a solution to address challenges across various scientific and industrial domains (Bensaude-Vincent, 2016). Its integration into agriculture and food industries holds the potential for improving both the quantity and quality of agricultural products while safeguarding environmental and natural resources. Nanotechnology, a relatively recent addition to agricultural practices, harnesses the unique physicochemical properties of atomic or molecular assemblies ranging from 1 to 100 nm in size (Monica and Cremonini, 2009).

The effective management of nutrient elements is of paramount importance for the successful cultivation of plants, as it affects both the quantity and quality of crop yields (Koch et al., 2020). Nanotechnology offers novel opportunities to enhance nutrient utilization efficiency in crops while minimizing associated environmental costs. For instance, potassium application has been shown to boost corn production, enhance crop quality, and increase plant resilience to salinity and drought, while also improving water and nutrient uptake efficiency (Amanullah et al., 2016). Potassium is essential for various metabolic functions in plants. It plays a critical role in enzyme reactions, respiration, absorption, and the stabilization of CO_2_. Additionally, it contributes to protein synthesis and affects photosynthesis by regulating stomatal opening. Furthermore, potassium enhances a plant’s ability to withstand environmental stress, such as drought. This is crucial for mitigating the negative effects of water scarcity (Johnson et al., 2022). Moreover, a higher concentration of potassium ions in mesophyll cells can improve water use efficiency, as demonstrated by recent research (Hu et al., 2022).

Potassium is an essential macronutrient for plants, influencing a multitude of physiological and biochemical processes. It regulates water status, turgor pressure, and the movement of photosynthates, thereby contributing to overall plant growth (Hasanuzzaman et al., 2018). In the context of abiotic stress, such as salt stress, K plays a role in maintaining ion homeostasis and osmotic balance. Furthermore, during drought stress, potassium plays a pivotal role in the opening of stomata, enabling plants to adapt to water deficits (Francini et al., 2022). Although silicon is not considered an essential element for plants, it has recently attracted significant interest in the field of agriculture due to its beneficial effects. It enhances the availability of nutrients, regulates transcription, and interacts with other essential and beneficial elements (Aqaei et al., 2020; Sattar et al., 2020; Malik et al., 2021; Rehman et al., 2021; Weisany and Razmi, 2023). Silicon plays a particularly prominent role in the context of stress-inducing conditions. Nevertheless, further research is required to elucidate its mechanisms and potential for sustainable agriculture (Aqaei et al., 2020; Sattar et al., 2020; Malik et al., 2021; Rehman et al., 2021; Weisany and Razmi, 2023).

Nanotechnology, particularly silicon nanoparticles (SiNP), offers a promising avenue for enhancing plant growth and resilience against biotic and abiotic stresses, offering a potential solution to the limitations of conventional methods. However, challenges remain due to the limited availability of plant-accessible (Aqaei et al., 2020; Sattar et al., 2020; Malik et al., 2021; Rehman et al., 2021; Weisany and Razmi, 2023). Numerous studies have highlighted the benefits of silica supplementation in enhancing resistance to environmental stresses, particularly drought stress (Aqaei et al., 2020; Sattar et al., 2020; Malik et al., 2021; Rehman et al., 2021; Weisany and Razmi, 2023). Optimal silica consumption levels can bolster cultivated plants’ tolerance to salinity and drought while improving soil water permeability (Aqaei et al., 2020; Rezakhani et al., 2022).

The use of nano-fertilizers, employing nano-structured or nano-scale materials as carriers or release controllers for fertilizers, holds promise in enhancing nutrient consumption efficiency. This innovation facilitates the development of smart fertilizers, leveraging nanotechnology to overcome current limitations associated with slow and controlled nutrient release (Subramanian et al., 2008). Over the past decade, numerous preliminary experiments have explored the potential of nanotechnology to enhance crop growth. Nanotechnology’s role in safeguarding agricultural products and food has been underscored by various studies emphasizing its environmental benefits. Its application in agriculture and related sectors holds promise for boosting productivity, refining product quality, and preserving global resources. Nano-fertilizers, for instance, offer a means to optimize nutrient utilization by regulating nutrient release from fertilizers (ul Ain et al., 2023). Silicon nanoparticles, too, have emerged as a significant contender in agriculture, potentially offering superior solutions for tackling diverse abiotic stresses compared to bulk materials (Cui et al., 2017; Tripathi et al., 2017).

The detrimental effects of drought stress on sorghum plants underscore the urgency of exploring novel strategies, such as potassium nanosilicate (PNS), to mitigate these effects. Despite the limited research on the effects of PNS on sorghum under drought stress in our country, this study aims to fill this gap by investigating the influence of PNS on sorghum growth and performance at the nanoscale. Our primary objective is to evaluate how PNS application affects sorghum growth, grain yield, and macro- and micronutrient uptake under drought stress conditions. Based on the existing literature and preliminary observations, we hypothesize that PNS application will lead to significant improvements in sorghum growth parameters, including plant height, biomass accumulation and reproductive yield under drought stress conditions. In addition, we expect PNS-treated sorghum plants to exhibit higher levels of macro- and micronutrient uptake compared to untreated plants, thereby mitigating the negative effects of drought-induced nutrient deficiencies. By conducting a comprehensive factorial experiment, we are breaking new ground and providing important insights into the potential of PNS to enhance crop resilience and productivity in the face of drought. This research not only fills a significant gap in current agricultural studies, but also contributes to the advancement of sustainable practices tailored to water-limited environments, paving the way for agricultural innovation and resilience.

## 2 Material and methods

### 2.1 Experimental design

An experiment was conducted to evaluate the influence of drought levels and PNS on the growth, grain yield, and uptake of macro and micronutrients in sorghum plants. The study was carried out during the 2021–2022 growing season at the research and education center of agriculture and natural resources in Al Madinah province. The experimental layout consisted of five rows, each extending 5 meters in length. Rows were spaced 75 cm apart, with a plant spacing of 20 cm within each row. Prior to planting, seeds underwent treatment with Vitawax fungicide. They were then sown in clusters of three at a depth of 3–5 cm to ensure robust establishment, with any excess seedlings removed at the 4–6 leaf stage.

### 2.2 Treatments

A factorial design was employed in the experiment, which utilized a complete randomized block layout with three replications. The first factor encompassed three levels of drought stress: non-stress (-0.03 MPa), moderate stress (-0.6 MPa), and severe stress (-1.2 MPa). The second factor comprised three concentrations of PNS, at concentrations of 0 mM (control), 3.6 mM Si, and 7.2 mM Si. The first factor encompassed three levels of drought stress, which were defined based on soil water potential measurements obtained using tensiometers. The three levels of drought stress were defined as follows: NS, MS, and SD. Throughout the experimental period, drought stress was applied and controlled using tensiometers and soil moisture sensors, which provided continuous monitoring of soil water potential. Tensiometers (Model SR Soil Moisture 24-Inch, USA) were installed at specific depths to accurately assess the soil matric potential, thereby ensuring an accurate representation of the water availability to the plants. The NS level represented optimal water conditions, where plants received sufficient irrigation to maintain near-field capacity. The MS level was achieved by reducing the irrigation frequency and quantity, while the SD level was achieved by further reducing the irrigation frequency and quantity. This process was designed to simulate realistic water deficit conditions.

At the six-to-eight leaf stage, a stock solution of 2.86 mM nano-Si and 10.2 mM nano-K was prepared for the treatment. The solution had a density of 1.05 g/cm³ and a pH of 7.4. A non-ionic surfactant (0.1% Tween 20) was included in the solution to ensure proper dispersion of nanoparticles. Subsequently, the PNS solution was diluted to final concentrations of 0 mM, 0.1 mM, and 0.2 mM and applied via foliar spraying at a rate of 3 L/h. The control group was administered an equivalent volume of distilled water containing the same concentration of surfactant. In order to maintain equilibrium of K in the treatment solution, an additional spray of potassium chloride (KCl) solution was performed. The KCl solution had a concentration of 10 mM K and was applied 30 minutes after the initial spraying of the nano-Si and nano-K solution in order to ensure optimal absorption. The control group was administered an equivalent volume of distilled water containing the same concentration of surfactant. In order to ensure optimal absorption of the PNS solution by the plants, a series of foliar sprays were conducted at three distinct stages: prior to flowering, during flowering, and at the grain filling stage.

**Figure 1:**
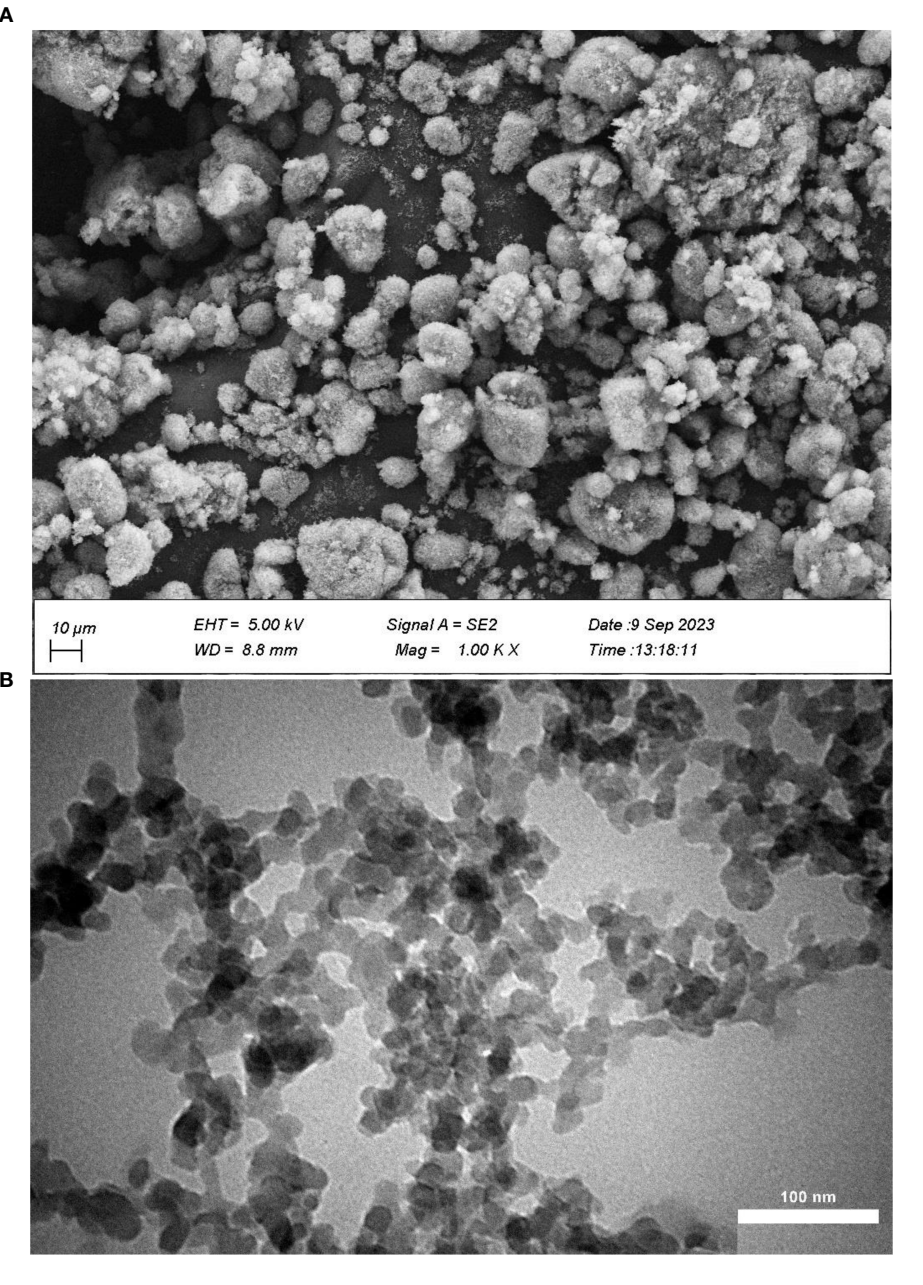
**(A)** scanning electron microscopy (SEM) and, **(B)** transmission electron microscopy (TEM) of potassium silica NPs.

Manual weeding was performed throughout the duration of the experiment, and seeds were harvested manually upon reaching full ripeness. In order to guarantee a consistent size distribution of the nanoparticles and to eliminate any unwanted substances, it is essential to implement selective precipitation based on size. This purification method is of paramount importance for enabling precise examination through scanning electron microscopy (SEM) and transmission electron microscopy (TEM). as shown in **Figure 1**. The SEM and TEM analysis, as illustrated in the diagram, indicates that the PNS particles have a size range of 20 to 100 nm, exhibit primarily spherical shapes, and have a fine surface texture. Some of the physico-chemical properties of the soil are shown in **Table 1**. At the time of the application of the spray, the temperature was approximately 25°C, and the relative humidity was approximately 60%. The spraying was conducted using a backpack sprayer equipped with a flat-fan nozzle. The sprayer operated at a pressure of 2 bar, ensuring uniform coverage of the foliage. A surfactant (0.1% Tween 20) was incorporated into the foliar applications with the objective of improving the adherence and spread of the solution on the plant leaves. In order to prevent the spray from reaching the soil, plastic shields were employed around the plants during the application process. This method effectively contained the spray within the target area, ensuring that only the foliage received the treatment.

**Table 1:** The soil physicochemical properties.

H_4_SiO_4_ (mg kg^-1^)	pH	EC (Ds m^-1^)	OC (%)	N (%)	P (mg kg^-1^)	K (mg kg^-1^)	Sand (%)	Silt (%)	Clay (%)
12.3	7.8	1.02	0.95	0.84	23.4	325	40	28	32

#### 2.2.1 Mineral nutrients

After harvest, shoot samples were dried in an oven set at 70°C for 48 hours. Following drying, the samples were ground into powder for each treatment group using an electric grinder. Nitrogen concentration in the shoot was determined through titration following distillation, employing the Kjeldahl method (Okalebo et al., 2002).

For phosphorus concentration analysis, a colorimetric method was utilized at a wavelength of 470 nm with a spectrometer. Plant samples underwent digestion in a flask containing sulfuric acid, salicylic acid, and hydrogen peroxide. The resulting extract was measured using a photometric method, specifically the yellow vanadate molybdate color, and a spectrophotometer (Jones et al., 1991).

Levels of potassium (K), calcium (Ca), and sodium (Na) were assessed using the dry digestion technique. Samples were dissolved in 0.5 M chloric acid and digested in an oven set at 550°C. Analysis was carried out using a film photometer device (Jones et al., 1991).

For micronutrient analysis, the dry ash method was employed. Firstly, the pulverized samples were dried in a 70°C oven. Then, dried leaves weighing 1 g were subjected to slow heating to 500°C in a ceramic container, resulting in white ash. Subsequently, each sample was mixed with 20 mL of 1-N HCl and left in a sand bath for 30 minutes after cooling to room temperature. Iron (Fe), copper (Cu), manganese (Mn), and zinc (Zn) were quantified using an atomic absorption spectrophotometer, model-2380 (Jones and Case, 1990).

### 2.3 Statistical analysis

A randomized complete block design was employed for the statistical analysis. The primary objective was to analyze the individual effects of nano compounds on the measured traits. This approach allows for a thorough examination of how each treatment influenced plant responses, ensuring robust and insightful findings. The research investigated the growth and nutrient uptake of sorghum plants under drought stress conditions with the application of PNS. A standard two-factor analysis of variance (ANOVA) was employed for the analysis. The data are expressed as mean ± standard deviation (SD) and were log-transformed to ensure normal distribution. The statistical analysis was conducted using SAS statistical software (version 9.3, SAS Institute, Cary, NC). Mean comparisons were conducted using Duncan’s Multiple Range test at a significance level of 5%, in accordance with the methodology outlined (Okalebo et al., 2002).

## 3 Result

**Figure 2:**
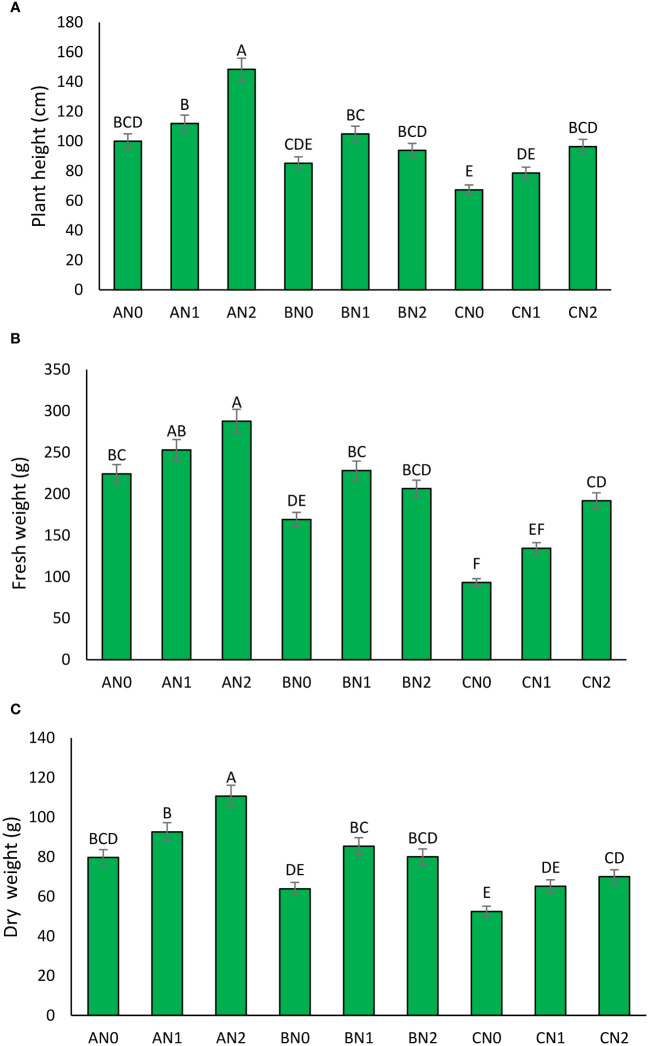
The effect of potassium nano-silica at 0 mM (N0), 3.6 mM (N1), and 7.2 mM (N2) levels, as well as different levels of drought stress - non-stress **(A)**, moderate **(B)**, and severe **(C)** - on plant height **(A)**, fresh weight **(B)**, and dry weight **(C)** of sorghum. Treatments that share a common letter do not show a significant statistical difference at the 5% probability level.

The impact of PNS on sorghum plant height under different drought stress conditions is evident. In the absence of stress, the plant height was observed to be 150 cm for the 0 mM treatment, 160 cm for the 3.6 mM treatment, and 170 cm for the 7.2 mM treatment (**Figure 2A**). In MD stress conditions, the plant height decreased to 120 cm for the 0 mM treatment, 130 cm for the 3.6 mM treatment, and 140 cm for the 7.2 mM treatment. In the context of severe drought stress, all treatments exhibited a significant reduction in plant height, with the 0 mM treatment reaching a height of 90 cm, the 3.6 mM treatment reaching 100 cm, and the 7.2 mM treatment reaching 110 cm. Notwithstanding the aforementioned reductions, the 7.2 mM treatment consistently yielded the highest plant height, thereby indicating the presence of some level of drought tolerance provided by the PNS (**Figure 2A**).

The application of PNS has been demonstrated to have a positive effect on fresh weight across all levels of drought stress (**Figure 2B**). In non-stress conditions, the fresh weight was 500 gr for the 0 mM treatment, 550 gr for the 3.6 mM treatment, and 600 gr for the 7.2 mM treatment. In the context of MD stress, the fresh weight exhibited a reduction to 400 gr for the 0 mM treatment, 450 gr for the 3.6 mM treatment, and 500 gr for the 7.2 mM treatment. In conditions of SD stress, all treatments exhibited a significant reduction in fresh weight, with the 0 mM treatment at 300 gr, the 3.6 mM treatment at 350 gr, and the 7.2 mM treatment at 400 gr. The 7.2 mM treatment consistently demonstrated a relatively higher fresh weight, indicating its efficacy in alleviating the effects of SD stress (**Figure 2B**).

The effect of PNS on dry weight exhibits a similar trend to that observed for plant height and fresh weight. In the absence of stress, the dry weight was 150 gr for the 0 mM treatment, 170 gr for the 3.6 mM treatment, and 190 gr for the 7.2 mM treatment (**Figure 2C**). Upon exposure to MD stress, the dry weight exhibited a decline, reaching 120 gr for the 0 mM treatment, 140 gr for the 3.6 mM treatment, and 160 gr for the 7.2 mM treatment. In SD stress conditions, all treatments exhibited a significant decline in dry weight, with the 0 mM treatment at 80 gr, the 3.6 mM treatment at 100 gr, and the 7.2 mM treatment at 120 gr. The 7.2 mM treatment consistently demonstrated the highest dry weight, indicating that PNS at this concentration may be effective in mitigating the adverse effects of SD stress on sorghum (**Figure 2C**).

**Figure 3:**
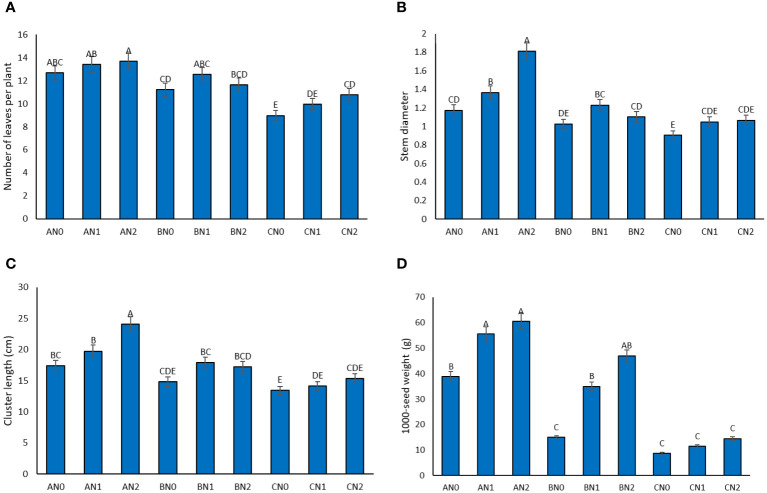
The effect of potassium nano-silica at 0 mM (N0), 3.6 mM (N1), and 7.2 mM (N2) levels, as well as different levels of drought stress - non-stress **(A)**, moderate **(B)**, and severe **(C)** - on leaf number **(A)**, stem diameter **(B)**, cluster length **(C)**, 1000-seed weight **(D)** of sorghum. Treatments that share a common letter do not show a significant statistical difference at the 5% probability level.

During drought stress, a reduction in the number of leaves per plant was observed. Plants subjected to NS exhibited the highest leaf count (13.25 leaves), while those under SD stress had the lowest leaf count (9.9 leaves) (**Figure 3A**). Analysis comparing the average number of leaves per plant with the influence of PNS revealed an increase compared to the control group. Notably, there was no significant difference between the 3.6 and 7.2 mM levels of PNS application.

Evaluation of the average stem diameter in plants under drought stress with PNS showed varying results (**Figure 3B**). The maximum stem diameter of 1.81 centimeters was recorded for plants treated with 7.2 mM PNS under MD, while the minimum stem diameter of 0.90 cm was observed in plants without PNS under SD. The most substantial effect of PNS in increasing stem diameter was observed in MD stress at 7.2 mM PNS and under control conditions at 3.6 mM PNS.

Under drought stress conditions, there was a significant reduction in the length of sorghum clusters. The sorghum plants displayed the longest cluster, measuring 20.39 cm under NS, while the shortest cluster, measuring 14.31 cm, was observed under SD drought stress conditions. **Figure 3C** illustrates the comparison of the average cluster length under the influence of MD stress and PNS. The results highlight a 14.3% improvement in cluster length under MD stress conditions with the application of 7.2 mM PNS.

The application of PNS significantly increased the dry weight of a thousand grains of sorghum under no drought stress conditions (**Figure 3D**). The highest weight of a thousand grains, 64.241 gr, was observed under NS, while the lowest weight, 188 gr, was recorded under stress conditions (SD) (**Figure 3D**). Furthermore, under MS conditions, the utilization of PNS resulted in an enhancement in the dry weight of a thousand grains in comparison to the absence of PNS. However, under SD stress conditions, the application of PNS did not have a significant effect on increasing the weight of the seeds (**Figure 3D**).

### 3.1 Nutrient

The results indicate that under drought stress, plants treated with NS exhibited the highest levels of N, while those treated with SD showed the lowest levels (**Table 2**). In non-stressful conditions, the application of PNS led to an increase in N content, with the highest value of 2.71% observed in the control group treated with 7.2 mM PNS (**Table 2**). However, under stress conditions, the application of PNS resulted in an improvement in N content, with values of 2.65% and 2.35% observed in the MD and SD treatments, respectively, in comparison to the control (**Table 2**). Furthermore, the phosphorus content of sorghum plants exhibited a notable decline under MD and SD stress conditions (**Table 2**). The application of PNS was found to mitigate this decrease, with P levels reaching up to 0.53% in the SD treatment in comparison to the control (**Table 1**).

**Table 2:** The effect of drought stress and potassium nano-silica on the macronutrient concentrations of sorghum seeds.

Drought stress	Potassium nano silica (mM)	Nitrogen (%)	Std	Phosphor (%)	Std	Potassium (%)	Std	Calcium (mg/kg)	Std	Sodium (mg/kg)	Std
A	N0	2.25b	0.13	0.42cd	0.03	1.15b	0.31	126.0ab	3.72	24.0ab	0.4
A	N1	2.67a	0.17	0.49bc	0.02	2.22ab	0.28	132.2ab	3.75	23.7ab	1.02
A	N2	2.71a	0.06	0.57a	0.01	2.85a	0.36	133.3a	7.97	25.5a	0.95
B	N0	2.14b	0.06	0.40cd	0.04	1.03c	0.29	125.9ab	1.83	22.2b	1.14
B	N1	2.65a	0.26	0.52ab	0.06	2.39a	0.31	126.2ab	1.44	23.7ab	1.02
B	N2	2.35b	0.11	0.51ab	0.06	2.23a	0.36	129.9ab	7.31	22.5b	0.69
C	N0	1.76c	0.04	0.48bc	0.05	0.79d	0.13	123.3ab	10.88	22.0b	1.11
C	N1	1.88c	0.14	0.51ab	0.04	2.19ab	0.16	120.0c	1.95	22.7b	1.78
C	N2	2.23b	0.16	0.53ab	0.02	2.38a	0.05	120.3c	9.73	22.7b	1.74

Means within one column sharing the same letter are not significantly different at 5% based on Duncan’s Multiple Range test. Std; standard deviation.

Potassium nano-silica at 0 mM (N0), 3.6 mM (N1), and 7.2 mM (N2) levels, Different levels of drought stress - non-stress (A), moderate (B), and severe (C).

The application of PNS resulted in a notable increase in the K content of sorghum plants, evident in both MD and SD stress conditions (**Table 2**). Specifically, plants treated with 7.2 mM PNS exhibited the highest K content, reaching 2.85%, while those without PNS application showed the lowest, at 0.79% (**Table 2**). Analysis of the data presented in **Table 2** highlights the significant impact of drought stress on the Ca content of sorghum plants. In both MD and SD condition, the Ca content was notably lower compared to the control, with values recorded at 125.9 mg/kg and 120.0 mg/kg, respectively. It is notable that the introduction of PNS did not yield a significant effect on the Ca content (**Table 2**). It is noteworthy that the introduction of PNS did not result in a discernible impact on the Ca content (**Table 2**). Moreover, while drought stress did not notably affect the sodium content of sorghum plants, there was a slight increase observed under non-stress (NS) conditions (25.5 mg/kg) and mild drought (MD) (23.7 mg/kg) in comparison to the control.

The Fe content in sorghum plants was significantly influenced by both drought stress and the application of PNS, as indicated in **Table 3**. Fe levels were notably lower under drought stress compared to the control treatment (**Table 3**). Application of PNS led to an increase in Fe content in sorghum plants under drought stress conditions. The highest Fe content, reaching 10.3 mg/kg, was observed in the NS treatment with 7.2 mM PNS, while the lowest, at 4.0 mg/kg, was recorded in the SD stress treatment without PNS application (**Table 3**). Likewise, Zn content exhibited significant sensitivity to drought stress, showing a noticeable decrease as drought stress levels intensified (**Table 3**). The application of 7.2 mM PNS resulted in a significant increase of 16.25 mg/kg and 15.52 mg/kg in both MD and SD stress in comparison to the control, respectively (**Table 3**). Furthermore, the Cu content of sorghum plants was also found to be significantly influenced by both drought stress and the application of PNS. In the context of moderate and severe drought conditions, respectively, Cu content exhibited a decline (**Table 3**). However, the application of PNS to sorghum plants subjected to MD (9.17 mg/kg) and SD (7.50 mg/kg) drought levels resulted in an increase in Cu content in comparison to the control (**Table 3**).

**Table 3:** The effect of drought stress and potassium nano-silica on the micronutrient and silica concentrations of sorghum seeds.

Drought stress	Potassium nano silica (mM)	Iron (mg/kg)	Std	Zinc (mg/kg)	Std	Copper (mg/kg)	Std	Manganese (mg/kg)	Std	Silica (mg/kg)	Std
A	N0	118.4b	5.4	16.62bc	0.93	8.49cbd	0.32	16.69b	1.4	4.83cd	0.55
A	N1	133.1a	10.3	19.13a	1.19	9.41ab	1.77	17.42ab	1.33	5.13cd	0.46
A	N2	134.7a	7.4	19.38a	1.36	9.98a	0.35	18.67a	1.17	6.76a	0.64
B	N0	100.4de	4.1	14.65cd	0.88	7.89cd	0.43	15.67bc	0.51	5.61bc	0.09
B	N1	117.7bc	7.7	17.75ab	1.45	9.17abc	0.59	16.50b	1.4	6.60a	0.12
B	N2	111.5cbd	10	16.26cbd	1.31	8.28cbd	0.25	16.93ab	0.56	6.15ab	0.56
C	N0	92.0e	4	13.95d	1.56	6.41ef	0.6	13.09d	0.64	3.45e	0.33
C	N1	106.9cbd	4.7	14.44cd	1.57	6.17f	0.6	13.73d	0.57	4.22de	0.76
C	N2	105.2cd	4.9	15.52cbd	1.77	7.50de	0.27	14.40cd	0.62	4.81cd	0.61

Means within one column sharing the same letter are not significantly different at 5% based on Duncan’s Multiple Range test. Std; standard deviation.

Potassium nano-silica at 0 mM (N0), 3.6 mM (N1), and 7.2 mM (N2) levels, Different levels of drought stress - non-stress (A), moderate (B), and severe (C).

Both MD and SD stress resulted in a significant reduction in Mn content in sorghum plants in comparison to the control group. The application of 7.2 mM PNS resulted in an increase in Mn content by 18.67, 16.93, and 14.40 mg/kg under NS, MD, and SD stress conditions, respectively (**Table 3**). The Si content of sorghum plants was significantly affected by drought stress and the application of PNS. In SD conditions without PNS, Si content decreased significantly by 3.45 mg/kg. However, the application of PNS on sorghum plants under drought stress resulted in an increase in Si content to 6.60 and 4.81 mg/kg under MD and SD stress conditions, respectively, in comparison to the control (**Table 3**).

## 4 Discussion

Under drought stress conditions, sorghum plants exhibit significant reductions in both plant height and leaf count per plant, ultimately leading to a decrease in both the fresh and dry weight of the plant. The onset of drought stress triggers leaf aging and subsequent shedding, further exacerbating the decline in leaf count (Aqaei et al., 2020). Given the pivotal role of leaves in photosynthesis and the synthesis of storage materials, it becomes evident that the decrease in plant growth under drought stress is partly attributable to the reduction in leaf number (Zhang et al., 2021). Insufficient photosynthesis, stemming from closed stomata and limited carbon dioxide absorption, represents another contributing factor to the diminished growth rate under stress conditions (McKersie and Lesheim, 2013). Moreover, drought stress reduces the turgor pressure of longitudinally growing stem cells, consequently decreasing the production of substances resulting from photosynthesis. This phenomenon leads to a reduction in the length of stem internodes and, consequently, the overall height of the plant, resulting in decreased plant weight (Abid et al., 2016).

The decrease in water potential of cells under drought stress induces plasmolysis, thereby diminishing the driving force necessary for cell growth (Tokarz et al., 2020). Furthermore, it leads to a reduction in the amount of water within plant cells and tissues. As soil moisture diminishes, protoplasm decay occurs alongside a reduction in cell mass, precipitating a sharp decline in cell size and division speed, ultimately resulting in decreased plant growth and photosynthetic activity (Zia et al., 2021). Consequently, the lack of water availability hampers cell growth and division, leading to decreases in both leaf number and area, as well as the height of the stem in the plant (Bhattacharya and Bhattacharya, 2021).

The results showed that the application of 7.2 mM PNS under SD stress conditions resulted in significant improvements in fresh weight (40.1%) of sorghum plants compared to no application of PNS under the same conditions. This indicates that PNS plays a pivotal role in facilitating plant growth under conditions of limited water availability. It is likely that this occurs by enhancing the availability and uptake of nutrients. The observed increase in plant height can be attributed to the enhanced availability of Si and K, which have been demonstrated to support cell elongation and division, as well as strengthen plant cell walls, thereby enhancing plant resilience to stress. These findings are consistent with previous research indicating that potassium plays a pivotal role in nutrient transport and absorption, thereby supporting the observed increase in seed yield (Sachin et al., 2019). The observed enhancements in various growth parameters under PNS treatment indicate an improvement in biomass accumulation. This can be attributed to the enhanced photosynthetic efficiency and reduced oxidative stress provided by Si and K. Moreover, the utilization of potassium has been associated with prolonged seed filling periods, leading to enhanced seed weight (Li et al., 2022). Additionally, potassium supplementation fosters favorable growth conditions, stimulates cell division, and promotes the synthesis of essential compounds like hydrocarbons and proteins, ultimately contributing to increased grain production (Tiwari et al., 2020). Furthermore, the incorporation of silica nanoparticles into plant roots has been found to fortify cell walls, bolstering the plant’s resilience against environmental stresses and consequently improving overall yield potential (Shinde et al., 2024). These combined effects underscore the multifaceted benefits of PNS in mitigating drought stress and enhancing crop productivity.

PNS significantly improved nutrient concentrations in sorghum seeds under drought stress, with notable improvements in K and Si, although statistical significance varied between treatments for other nutrients such as P and Ca. This reduction is attributed to a number of factors, including reduced soil moisture, impaired root development, decreased nutrient absorption rates, shorter total root length, and smaller root absorption areas (Cakmak, 2008).These factors collectively contribute to slower growth rates and decreased biomass accumulation in plants under drought conditions due to impaired nutrient uptake and assimilation. The mechanisms underlying this diminished nutrient uptake under drought stress encompass several factors. Firstly, soil moisture deficiency impairs nutrient solubility and mobility. Secondly, impaired root development constrains the plant’s capacity to explore the soil for nutrients. Thirdly, reduced hydraulic conductivity hinders nutrient transport from roots to shoots. Fourthly, diminished photosynthetic activity due to lower nutrient levels, particularly nitrogen and phosphorus, results in reduced carbohydrate production and energy availability for growth.

Drought stress significantly impedes the uptake of N by plants. This decline in N concentration under drought conditions can be attributed to diminished N transport via mass flow, stemming from inadequate moisture levels. Consequently, the transfer of nitrate and ammonium ions to the root surface diminishes, resulting in reduced N uptake and lower concentration levels (Zorb et al., 2014). The mobility of N is severely constrained in dehydrated soil during prolonged drought periods, leading to N deficiency in plants (Skalski et al., 2024). Drought stress hampers cellular and tissue development, reduces the absorption of vital nutrients, and triggers various morphological and biochemical alterations (Iqbal et al., 2020). Initially, drought may impede water uptake from the upper soil layers, which typically harbor higher soil nutrient concentrations, before affecting deeper soil layers (Ma et al., 2024). Drought-induced limitations on water uptake can result in diminished plant absorption of nutrients like P (Rea et al., 2022). In soils where P levels are already deficient, this exacerbates stress on both plants and ecosystems, given P’s crucial role in enhancing water-use efficiency and regulating stomatal function. Additionally, drought conditions have the potential to impede nutrient uptake kinetics per unit root, possibly by suppressing the activity of enzymes involved in nutrient assimilation, thereby slowing nutrient uptake (Robredo et al., 2011). Moreover, reduced transpiration due to drought diminishes volume flow in the xylem, constraining transport and reducing mass flow and diffusivity of nutrients between roots and stems (Liang et al., 2018).

Application of PNS improved nutrient absorption, particularly of K and Si, during drought stress. K enhances the translocation of photosynthesis products, thereby promoting better plant growth (Rawat et al., 2022). The addition of Si to soil during plant cultivation is likely to increase P uptake, as Si has been shown to enhance P absorption in shoots. This study aligns with previous research indicating that Si supplementation increases P uptake (Kostic et al., 2017; Neu et al., 2017). Under drought stress conditions, PNS application resulted in increased K content, with a more pronounced increase observed at higher doses. This enhancement could be attributed to the capacity of K to stimulate root development and improve nutrient absorption (Sustr et al., 2019). It appears that PNS alters the metabolism of absorbed K in plants, promoting its conversion into amino acids and proteins by stimulating carbohydrate production (Chieb and Gachomo, 2023). The increased K absorption may be linked to Si-mediated reductions in plasma membrane permeability and Si-induced enhancements in plasma membrane H^+^-ATP activity (Kaya et al., 2006). Additionally, Si may augment nutrient uptake by boosting root activity (Chen et al., 2011).

Adequate nutrient supply is of paramount importance for the enhancement of both the quality and quantity of plant growth. K is one of the essential nutrients required for optimal plant functioning. The presence of K in plants enhances their resilience to environmental stress, while facilitating the synthesis of starch and carbohydrates. It is indispensable for maintaining CO_2_ stability in chloroplasts and for the ribosomal activities of carboxylase bisphosphate. In cases of K deficiency, enzyme activities such as synthesis, transferases, and kinases are disrupted, leading to reduced ATPase activity and impeding the absorption and transfer of certain nutrients (Kanai et al., 2007). Increasing K concentration boosts photosynthetic product formation, regulates ionic balance, facilitates osmotic adjustment, regulates stomatal movement, and enhances enzyme activity. Application of PNS promotes greater nitrogen absorption by activating various enzymes such as ADP-glucose synthesis and participating in physiological processes, thereby enhancing plant resistance to environmental stress. K plays a pivotal role in carbohydrate production (Sardans and Peñuelas, 2021).

The interactions among nutrients can impact nutrient absorption processes and plant functioning, potentially leading to the production of toxins that can hinder plant growth (Ashkavand et al., 2018). Increased Si application has been shown to enhance nutrient absorption in rice seeds and shoots (Kalyan Singh et al., 2006). Application of nano-sized Si spray has been found to enhance growth and the absorption of Zn, Fe, and Mn in rice plants (Wang et al., 2015). Si nutrition contributes to improved root growth, development, and weight, consequently enhancing absorption levels, consistent with findings reported by (Alsaeedi et al., 2019). One potential explanation for the observed increase in the concentration of certain elements in plants is the promotion of their binding within plant tissues by Si. Furthermore, Si has been observed to influence the translocation of elements to the shoot, as evidenced by the case of Zn and Cu. Furthermore, Si can influence the development of apoplasmic barriers in roots, regulating apoplasmic pathways and subsequent translocation via root apoplasm to the shoot (Vaculíková et al., 2016). Si can alleviate water stress by reducing transpiration, which is mediated through stomata and the cuticle. Rice plants typically have a thin cuticle, and the formation of a cuticle-Si double layer significantly reduces cuticular transpiration. Consequently, silicon not only influences the availability and uptake of nutrients but also affects the translocation of nutrients from roots to shoots (Ali et al., 2020).

The application of PNS has been demonstrated to mitigate the adverse effects observed, enhancing nutrient uptake and plant growth through a number of mechanisms. The application of PNS has been demonstrated to support better root growth and function under drought conditions. This is evidenced by increases in root length and absorption area, improvements in soil moisture retention, and enhancements in nutrient solubility and mobility. Furthermore, the presence of silicon and potassium in PNS stimulates the plant’s stress defense mechanisms, activating antioxidant enzymes that reduce oxidative damage and support overall plant health. This enables better nutrient assimilation and utilization. The improvements in nutrient uptake observed in plants treated with PNS resulted in enhanced biomass accumulation and growth parameters. The enhanced availability of essential nutrients facilitates vital physiological processes, resulting in accelerated growth rates and elevated biomass production, even in the presence of drought stress.

## 5 Conclusions

When sorghum plants were subjected to drought stress and treated with potassium nanosilicate (PNS), significant improvements in several growth parameters were observed. While our results show increases in essential elements such as nitrogen (N), potassium (K) and silicon (Si) in seeds and nitrogen (N), potassium (K) and copper (Cu) in plants, the statistical analyses of the data presented highlight these improvements without fully supporting the conclusion that PNS improves physical characteristics and yield or strengthens plant resistance to drought challenges. Nevertheless, our study represents a novel exploration of PNS’s potential to mitigate nutrient deficiencies exacerbated by drought stress, highlighting its role in sustainable crop management. Future research should further investigate the mechanisms and long-term effects of PNS on soil health and crop productivity, and explore synergies with other agricultural strategies to optimize its effectiveness in mitigating drought stress and ensuring food security in the face of environmental challenges.

## Data availability statement

The raw data supporting the conclusions of this article will be made available by the authors, without undue reservation.

## Acknowledgements

Princess Nourah bint Abdulrahman University Researchers Supporting Project number (PNURSP2024R188), Princess Nourah bint Abdulrahman University, Riyadh, Saudi Arabia.

## Author contributions

KA: Writing – original draft, Writing – review & editing. GS: Writing – original draft, Writing – review & editing. TS: Writing – original draft, Writing – review & editing. SM: Writing – original draft. IA: Writing – original draft. AA: Writing – original draft, Writing – review & editing. MH: Writing – original draft, Writing – review & editing.
